# Endothelium-Independent Vasorelaxant Effects of Hydroalcoholic Extract from *Nigella sativa* Seed in Rat Aorta: The Roles of Ca^2+^ and K^+^ Channels

**DOI:** 10.1155/2014/247054

**Published:** 2014-05-12

**Authors:** Saeed Niazmand, Elahe Fereidouni, Maryam Mahmoudabady, Seyed Mojtaba Mousavi

**Affiliations:** ^1^Cardiovascular Research Center, School of Medicine, Mashhad University of Medical Sciences, Mashhad 9177948564, Iran; ^2^Department of Physiology, School of Medicine, Mashhad University of Medical Sciences, Mashhad 9177948564, Iran; ^3^Neurogenic Inflammation Research Centre, School of Medicine, Mashhad University of Medical Sciences, Mashhad 9177948564, Iran

## Abstract

*Objective.* The aim of this study was to elucidate the mechanism(s) responsible for the vasorelaxant effect of *Nigella sativa* (*N. sativa*). *Methods.* The activity of different concentrations of *N. sativa* extract was evaluated on contractile responses of isolated aorta to KCl and phenylephrine (PE). *Results.* The extract (2–14 mg/mL) induced a concentration dependent relaxation both in endothelium-intact and endothelium-denuded aortic rings precontracted by PE (10^−6^ M) and KCl (6 × 10^−2^ M). Extract reduced PE- and KCl-induced contractions in presence of cumulative concentrations of calcium (10^−5^–10^−2^ M) significantly. L-NAME and indomethacin had no effect on vasorelaxation effect of extract in PE-induced contraction. Diltiazem and heparin reduced significantly this vasorelaxation at a concentration of 14 mg/mL of extract; however, *N. sativa*-induced relaxation was not affected by ruthenium red. Tetraethylammonium chloride reduced the extract-induced relaxation in concentrations of 2–6 mg/mL of extract significantly but glibenclamide reduced this relaxative effect in all concentrations of extract. *Conclusions.* The inhibitory effect of *N. sativa* seed extract on the contraction induced by PE and KCl was endothelium-independent. This relaxation was mediated mainly through the inhibition of Ca^2+^ and K_ATP_ channels and also intracellular calcium release.

## 1. Introduction

*Nigella sativa (N. sativa)* which is commonly known as black cumin is a plant from the Ranunculaceae (buttercup) family. This plantisnative to Southern Europe, North Africa, and Southwest Asia and is cultivated in many countries in the world like those in the Middle Eastern Mediterranean region, Southern Europe, India, Pakistan, Iran, Syria, Turkey, and Saudi Arabia [1].

The seeds of* N. sativa* are used in folk (herbal) medicine all over the world for the treatment and prevention of a number of diseases and conditions that include asthma, diarrhea, and dyslipidemia [2].* N. sativa* has been extensively studied for its biological activities and therapeutic potential and has been shown to possess a wide spectrum of activities such as anti-inflammatory [3], reduced ischemia-reperfusion injury [4], antioxidant [5, 6], antiepileptic [7], antibacterial [8], antihistaminic [9], antinociceptive [10], antidiabetic [11], hepatoprotective [12], and smooth muscle relaxant [9, 13] effects.

The seed oil of* N. sativa* was found to be rich in polyphenols and tocopherols [14, 15]. The seeds contain 36–38% fixed oils, 0.4–2.5% essential (volatile) oil, proteins, alkaloids, and saponins. The fixed oil is composed mainly of fatty acids, namely, linoleic (C18:2), oleic (C18:1), palmitic (C16:0), and stearic (C18:0) acids [16]. Thymoquinone (TQ) is the most pharmacologically active ingredient found abundantly (30–48%) in the black seeds, together with its derivatives such as dithymoquinone, thymohydroquinone, and thymol [17].

There is increasing evidence of the cardiovascular effect of* N. sativa* such asantihypertensive [18–21], hypotensive [22, 23], antihyperlipidemic [24–27], and ameliorative effect of endothelial dysfunction [28, 29]; however, the vasorelaxant effect of* N. sativa* was not evaluated. Thus, the present study investigated the effects of hydroalcoholic extract of* N. sativa* seed on the vasomotor tone of the aortic rings and its possible mechanism(s) of action.

## 2. Materials and Methods

### 2.1. Chemicals and Drugs

All chemicals were of analytical grade (Merck). Phenylephrine hydrochloride (PE), acetylcholine (ACh), N^G^-nitro-L-arginine methyl ester (L-NAME), indomethacin, ruthenium red (RR), heparin (HP), tetraethylammonium chloride (TEA), and diltiazem were obtained from Sigma (Germany). Moreover, when necessary, the Krebs solution was used as solvent for all drugs.

### 2.2. Plant Material and Preparation of the Extract

*N. sativa* was collected from Nishabour city, Khorasan Province, Iran, and its seeds were dried at room temperature in the absence of sunlight. The plant was identified by botanists in the herbarium of the Ferdowsi University of Mashhad; the specimen number of the plant is 176-2013-9. The hydroethanolic extract was prepared using a maceration method as follows: 500 g of chopped* Nigella sativa* seeds were soaked in 500 cc of 50% ethanol for 48 hours at room temperature and the mixture was subsequently filtered and concentrated* in vacuo* at 40°C. The residue was suspended in saline solution to obtain 2, 4, 6, 8, 10, and 14 mg/mL concentrations.

### 2.3. Experimental Animals

Fourteen groups of Wistar rats (200 to 250 g, *n* = 8 for each group) were studied. The animals were group-housed in cages at 22 ± 2°C temperature and given water and food* ad libitum*, while a 12 h on/12 h off light cycle was maintained. All experiments were conducted in accordance with the internationally accepted principles for laboratory animal use and care and with institutional guidelines.

### 2.4. Preparation of Rat Aortas

The animals were anesthetized with 50 mg/kg Ketamine and decapitated by guillotine; after thoracotomy, the descending thoracic aorta was exposed and was rapidly dissected out and immersed in 95% O_2_/5% CO_2_-gassed (carbogen) ice-cold Krebs solution with the following composition (mM): NaCl (118.5), KCl (4.74), MgSO_4_ (1.18), NaHCO_3_ (24.9), KH_2_PO_4_ (1.2), CaCl_2_ (2.5) and glucose (10), pH = 7.4. Aorta was then dissected free of periadventitial fat and connective tissue, with care taken to avoid touching the luminal surface and cut into four rings, each 5 mm in length. The aortic rings were mounted in 10 mL organ bath containing Krebs solution gassed with carbogen at 37°C. After a resting tension of 2 g, the vessel segments were allowed to equilibrate for 1 hour. Changes in tension were recorded by isometric transducers connected to a data acquisition system (AD instrument, Australia). In some rings, the endothelium was denuded by gently rubbing the intimal space with a thin metal rod. The absence of functional endothelium was verified by the inability of ACh (10^−5^ M) to induce the relaxation of rings precontracted with PE (10^−6^ M).

### 2.5. Experimental Procedure

#### 2.5.1. Effect of* N. sativa* Extract on Aortic Contraction Induced by PE and KCl

These experiments were made to verify* N. sativa* extract induced relaxation effect. A steady contraction in rings with the endothelium intact or denuded was induced by 10^−6^ M PE or 6 × 10^−2^ M KCl, and* N. sativa* was added cumulatively (2, 4, 6, 8, 10, and 14 mg/mL). The* N. sativa* extract induced relaxation in the aortic rings which was calculated as a percentage of the relaxation in response to PE and KCl.

#### 2.5.2. *N. sativa* Extract Induced Relaxation, L-NAME, and Indomethacin

To determine the nitric oxide (NO) or prostacyclin mediated relaxant effect of* N. sativa*, aortic rings were rinsed and exposed to L-NAME (10 *μ*M), a nitric oxide synthase inhibitor, or indomethacin (10 *μ*M), a cyclooxygenase (COX) inhibitor, for 30 min before induction of a steady contraction by 10^−6^ M PE, and final effects of cumulative concentrations of* N. sativa* extract (2, 4, 6, 8, 10, and 14 mg/mL) were evaluated for 25 minutes.

#### 2.5.3. *N. sativa* Extract Induced Relaxation, Influx of Ca^2+^, and Ca^2+^ Channels

In the first set of these experiments, an attempt was made to verify the relaxation induced by* N. sativa *involving Ca^2+^ influx. The endothelium-denuded aortic rings were washed four to five times with Ca^2+^-free Krebs solution (containing 5 × 10^−5^ M EGTA) before PE (10^−6^ M) or KCl (6 × 10^−2^ M) was applied to produce a steady contraction, and then Ca^2+^ was added cumulatively to obtain a concentration-response curve (10^−5^ to 10^−2^ M) in the presence of 14 mg/mL* N. sativa* extract. In the second set of experiments, the aim was to evaluate the roles of voltage-dependent calcium channels in extract induced relaxation. Endothelium-denuded aortic rings were exposed to diltiazem (10^−5^ M), an L-type Ca^2+^ channel inhibitor, for 30 min before the application of PE (10^−6^ M) to induce a steady contraction; subsequently, the* N. sativa* extract (14 mg/mL) was added to evoke a relaxation.

#### 2.5.4. *N. sativa* Extract Induced Relaxation and Intracellular Sources of Ca^2+^

In this set of experiments, the aim was to clarify whether the relaxation induced by* N. sativa* was related to the inhibition of intracellular Ca^2+^ release.

Endothelium-denuded aortic rings were exposed to diltiazem (10^−5^ M), an L-type calcium blocker, for 30 min before the application of PE (10^−6^ M) to induce a steady contraction; subsequently, the* N. sativa* extract (14 mg/mL) was added to evoke relaxation. In the presence of diltiazem, ruthenium red (RR) (10^−5^ M), a ryanodine receptor inhibitor, or heparin (HP) (50 mg/L), an IP_3_ receptor inhibitor, was added 30 min before the application of PE in separate experimental groups.

#### 2.5.5. *N. sativa* Extract Induced Relaxation and K^+^ Channels

To examine the role of K^+^channels in the extract induced relaxation, the aortic rings were rinsed and exposed to glibenclamide (10^−5^ M), an inhibitor of the ATP-dependent K^+^channels (K_ATP_), and tetraethylammonium chloride (TEA) (5 mM), a nonselective K^+^ channel blocker, for 30 min before the application of 10^−6^ M PE to induce a steady contraction and finally the effects of cumulative concentrations of the extract (2, 4, 6, 8, 10, and 14 mg/mL) were evaluated for 25 min.

### 2.6. Data Analysis

All data are expressed as mean ± S.E.M. The EC_50_ was defined as the concentration of* N. sativa* that induced 50% of the maximum relaxation from the contraction elicited by PE (10^−6^ M) or KCl (6 × 10^−2^ M) and was calculated from the concentration-response curve, analyzed by nonlinear regression (curve fit) using GraphPad Prism (Version 4.0). Statistical comparisons were made using the Student's* t*-test and one-way ANOVA followed by the Tukey's test. *P* values less than 0.05 were considered to be statistically significant.

## 3. Results

### 3.1. Effect of* N. sativa* on PE and KCl Contracted Aorta

**Figure 1:**
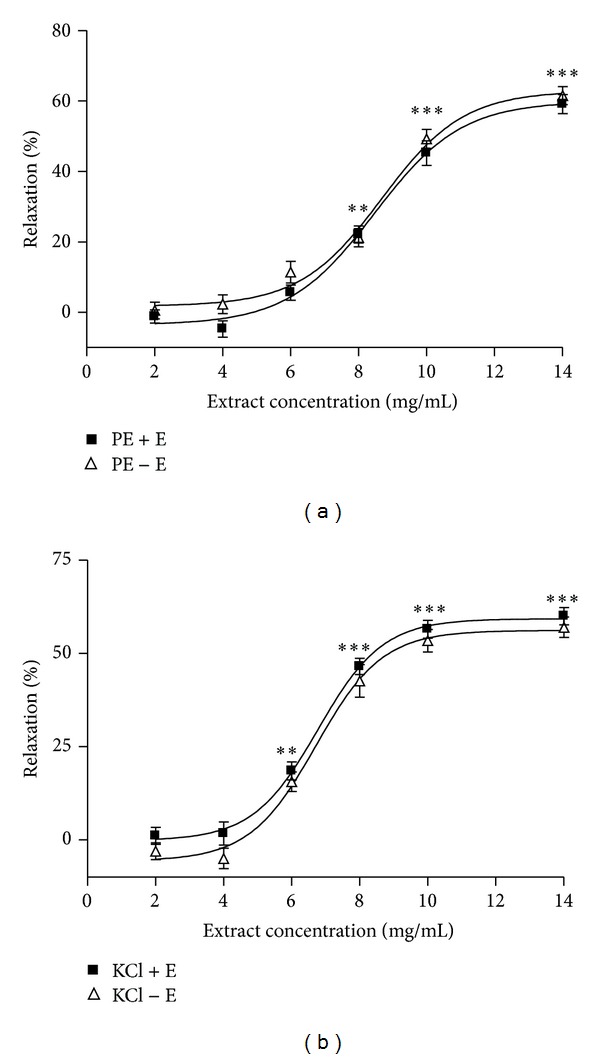
Effect of different concentrations of* Nigella sativa* extract (2, 4, 6, 8, 10, and 14 mg/mL) on PE (10^−6^  M) (a) and KCl (6 × 10^−2^  M) (b) precontracted rat aortic rings with (+E) or without (−E) endothelium. Data are expressed as mean ± S.E.M. (*n* = 8). ***P < 0.001, **P < 0.01 compared to base.

The* N. sativa* extract induced concentration-dependent relaxation in aortic rings precontracted by PE and KCl with a maximum relaxation of 62.3 ± 1.9% (EC_50_ = 8.5 mg/mL) and 60.2 ± 1.2% (EC_50_ = 7.6 mg/mL), respectively (Figures 1(a) and 1(b)). These inhibitory responses of extract were not significantly different in the intact and denuded aortic rings.

### 3.2. Effect of L-NAME and Indomethacin on Relaxant Response of* N. sativa*

**Figure 2:**
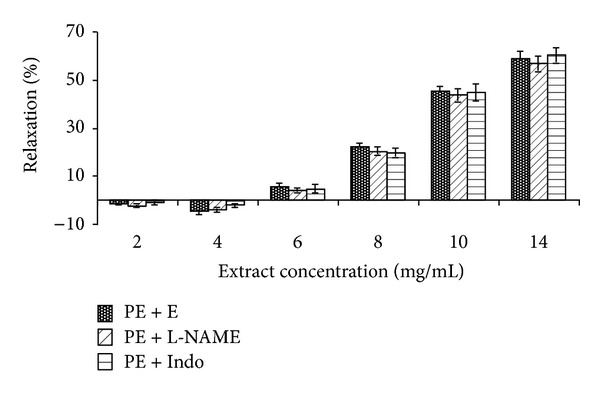
Effect of cumulative concentrations of* Nigella sativa* seed extract (2, 4, 6, 8, 10, and 14 mg/mL) on PE precontracted rat aortic rings with endothelium (PE + E) and after pretreatment with L-NAME (10 *μ*M) (PE + L-NAME) or indomethacin (10 *μ*M) (PE + Indo). Data are expressed as mean ± S.E.M. (*n* = 8).

Pretreatment of endothelium-intact aortic rings with L-NAME and indomethacin had no effect on the* N. sativa*-induced vasorelaxation at any concentration of extract (Figure 2).

### 3.3. Effect of* N. sativa* on Extracellular Ca^2+^-Induced Contraction

**Figure 3:**
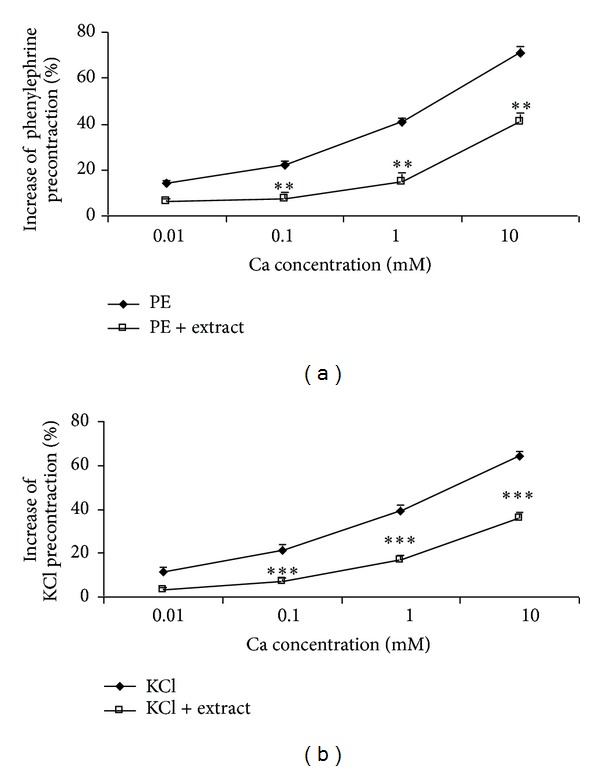
Effect of* Nigella sativa* seed extract at 14 mg/mL on the Ca^2+^-induced (0.01–10 mM) contraction of rat aortic rings without endothelium pretreated with PE (10^−6^ M) (a) or KCl (6 × 10^−2^ M) (b). Data are expressed as mean ± S.E.M. (*n* = 8). **P < 0.01, ***P < 0.001 compared tocontrol.

Cumulative addition of Ca^2+^ in a Ca^2+^-free medium containing PE or KCl induced a concentration-dependent contraction of aortic rings. Preincubation of the rings with 14 mg/mL of* N. sativa* significantly inhibited Ca^2+^-induced contraction in both PE (Figure 3(a)) and KCl (Figure 3(b)) constricted rings.

### 3.4. Effect of* N. sativa* on Intracellular Sources of Ca^2+^

**Figure 4:**
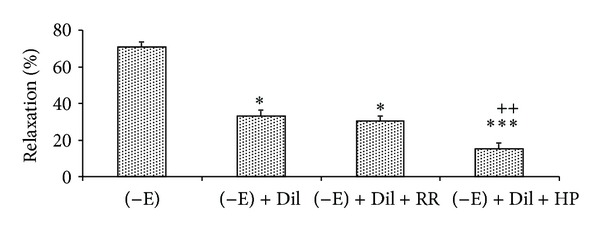
Effect of* Nigella sativa* seed extract (14 mg/mL) on endothelium-denuded rat aortic rings (−E) contracted with PE (10^−6^ M), in the presence of diltiazem (10^−5^ M) (−E + Dil), after ruthenium red (10^−5^ M) (−E + Dil + RR) or heparin (50 mg/L) (−E + Dil + HP) pretreatment. Data are expressed as mean ± S.E.M. (*n* = 8). *P < 0.05, ***P < 0.001 compared to −En; ^++^ *P* < 0.01 compared to −E + Dil.

The results of 30 min preincubation of endothelium-denuded aortic rings with RR or heparin in the presence of diltiazem with subsequent contraction by PE showed that diltiazem attenuated* N. sativa*-induced vasorelaxation in concentration of 14 mg/mL and RR did not change this reduction; however, heparin significantly diminished this effect of the extract (Figure 4).

### 3.5. Effect of* N. sativa* on K^+^Channels

**Figure 5:**
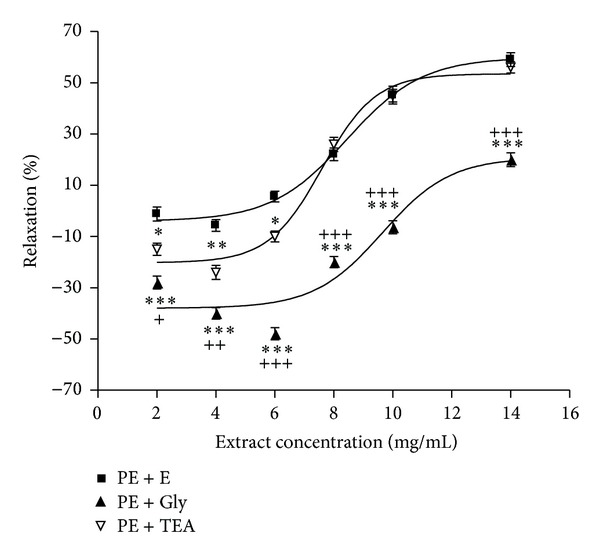
Effect of cumulative concentrations of* Nigella sativa* seed extract (2, 4, 6, 8, 10, and 14 mg/mL) on rat intact aortic rings contracted with PE (10^−6^ M) (PE + E), after pretreatment with glibenclamide (10^−5^ M) (PE + Gly) or tetraethylammonium chloride (5 mM) (PE + TEA). Data are expressed as mean ± S.E.M. (*n* = 8). *P < 0.05, **P < 0.01, and ***P < 0.001 compared to PE + E; ^+^ *P* < 0.05, ^++^ *P* < 0.01, and ^+++^ *P* < 0.001 compared to PE + TEA.

30 min preincubation of intact aortic rings with glibenclamide or TEA with a subsequent contraction by PE showed glibenclamide significantly reduced* N. sativa*-induced relaxation in all concentrations of extract but TEA reduced this relaxative effect only in concentrations of 2, 4, and 6 mg/mL (Figure 5).

## 4. Discussion

The results of the present study showed that* N. sativa* seed extract elicits vasorelaxation in aortic rings contracted by KCl and PE. Vasocontraction or vasorelaxation could be dependent on endothelium productions [30]. Endothelium through the production of substances such as nitric oxide (NO) and prostacyclin inhibits contraction and by secretion of endothelin can cause contraction in vascular smooth muscle cells (VSMCs) [31, 32].

The lack of variation between the vasorelaxation induced by* N. sativa* seed extract in intact- or denuded-endothelium of aortic rings suggests that this relaxant effect has been exerted on the VSMCs and not on the aorta endothelium (Figure 1).

The inhibitory effect of the extract on PE-induced contraction was not affected by the presence of L-NAME and indomethacin. NO and prostacyclin, the important factors of the vascular relaxant, are derived from the endothelium. Relaxant effect of NO is mainly due to an increase in cyclic guanosine monophosphate (cGMP). L-NAME as an inhibitor of NO production and indomethacin as a nonselective inhibitor of COX had no effects on the vasorelaxant effect of the extract on PE-induced contractions, which indicated that the relaxant effect of the extract is dependent on neither NO nor prostacyclin (Figure 2). Also, the absence of difference between intact- or denuded-endothelium of aortic rings in extract induced vasorelaxation confirms the independency of this extract's effect on the endothelium. Moreover, the inhibitory effects of the extract on the contraction induced by KCl and PE in intact- and denuded-endothelium of aortic rings were not different which is in agreement with this recent claim.

Ca^2+^ is a critical factor in the excitation-contraction coupling in smooth muscle cells [33, 34]. Influx of extracellular Ca^2+^ through receptor-operated Ca^2+^ channels (ROCCs) and voltage-dependent Ca^2+^ channels (VDCCs) and release of Ca^2+^ from the sarcoplasmic reticulum by activation of 1,4,5 triphosphate inositol (IP_3_) and ryanodine receptors (RYR) [35–37] result in increased intracellular Ca^2+^, which causes contraction. On the other hand, the contraction elicited by KCl mainly results from the influx of extracellular Ca^2+^ induced by depolarization of the cell membrane and subsequent opening of the VDCCs [36].

PE, an adrenoreceptor agonist, causes aortic contraction by Ca^2+^ influx through ROCCs and by release of Ca^2+^ from the sarcoplasmic reticulum [37, 38]. The latter pathway involves PE stimulation of phospholipase C to produce diacylglycerol (DG) and IP3, and subsequently DG activates the light chain of myosin through activation of protein kinase C (PKC), and IP3 induces Ca^2+^ release from the sarcoplasmic reticulum by opening IP3 receptors [37]. In our experiments,* N. sativa* seed inhibited the contraction of aortic rings induced by PE, implying that* N. sativa* seed may inhibit the IP_3_ and/or ryanodine receptor-dependent release of intracellular Ca^2+^, reduce DG-PKC dependent myosin light chain kinase activity, and/or block ROCCs to decrease intracellular Ca^2+^ and relax the aorta. And, the finding that* N. sativa* seed reduced the aortic contraction when PE produced a steady contraction followed by gradual Ca^2+^ input in a Ca^2+^-free solution, indicated that* N. sativa* seeds blockade of ROCCs to decrease the influx of extracellular Ca^2+^ may be a critical mechanism in relaxing the aorta.

Potassium chloride commonly causes a sustained contraction in isolated arteries.* N. sativa* seed extract had a concentration-dependent relaxant effect on this contraction which may be due to the effects on VDCCs in the VSMCs.

Results from previous studies have shown that the VDCCs are involved in KCl-induced contraction, so the inhibitory effects of vasorelaxant substances which affect this type of contractions may be through blocking VDCCs [33]. The relaxant effects of* N. sativa* seed extract on KCl-induced contraction in aortic rings in presence of cumulative concentrations of calcium are similar to the function of VDCCs in this suppressing effect.

To understand the effect of* N. sativa* seed on extracellular Ca^2+^ influx, experiments were conducted on rings contracted with PE or KCl in a Ca^2+^-free Krebs solution in which Ca^2+^ was added subsequently. Our data reporting that* N. sativa* seed decreased Ca^2+^-induced contractions after both PE- and KCl-induced contraction argue for the blockade of both ROCCs and VDCCs as part of the vasodilating effects of* N. sativa *seed. These results were verified by PE- or KCl-induced contraction in the presence of diltiazem as an L-type calcium blocker, in which the vasorelaxant effect of* N. sativa* seed decreased significantly (Figures 3(a) and 3(b)).

Relaxant effect of the extract was reduced significantly in the presence of heparin as an IP_3 _receptor inhibitor, which shows the importance of IP_3_ signaling pathway in the relaxant effect of* N. sativa* seed.

Ruthenium red did not diminish the extract induced vasorelaxation in aortic rings precontracted by PE; thus, the ryanodine receptors did not have any role in the inhibitory effect of the extract.

Besides Ca^2+^ channels, K^+^ channels contribute to the regulation of the membrane potential in electrically excitable cells including VSMCs [39]. Membrane hyperpolarization is due to an efflux of K^+^ rises of the opening of the K^+^ channels in the VSMCs. This effect is followed by the closure of VDCCs, leading to the reduction in Ca^2+^ entry and vasodilation [36]. VSMCs express both K_ATP_ and nonselective K^+^ channel [40, 41]. Blockade of the K_ATP_ channel by glibenclamide significantly decreased the relaxant effects of the extract which confirmed the prominent role of these K^+^ channels in the* N. sativa* seed induced vasorelaxation. Reduced inhibitory effect of the extract at concentrations of 2, 4, and 6 mg/mL by TEA showed implication of nonselective K^+^ channel in the* N. sativa* seed induced vasorelaxation at lower concentrations of extract.

These results suggest that the relaxant effects of* N. sativa* seed extract on the contractions induced by PE and KCl in VSMCs are mediated by different signaling pathways. It seems that the most important mechanisms involved in this vasorelaxation are inhibition of extracellular Ca^2+^ influx, blockade of K_ATP_ channels, and also suppression of IP_3_-mediated receptors. The previous study showed that cardiac inhibitory effect of* N. sativa* seed may be due to calcium channel inhibitory or an opening effect for the plant on potassium channels in isolated heart [42].

## 5. Conclusions

Based on the present data, the use of* N. sativa* seeds may be useful in traditional medicine for hypertension treatment that supports the previous studies which showed the antihypertensive effect of this plant.
